# Does Thyroidectomy Impact Quality of Life: Retrospective Case–Control Study of Post-Thyroidectomy Patients and Matched Individuals from the General Population

**DOI:** 10.3390/medicina56110603

**Published:** 2020-11-10

**Authors:** Hyeong Won Yu, Ah Reum An, Hye In Kang, Yong Joon Suh, Hyungju Kwon, Su-jin Kim, Young Jun Chai, June Young Choi, Hochun Choi, Kyu Eun Lee, Belong Cho

**Affiliations:** 1 Department of Surgery, Seoul National University Bundang Hospital, Seongnam-si 13620, Korea; hyeongwonyu@gmail.com (H.W.Y.); aznagran@gmail.com (J.Y.C.); 2 Department of Family Medicine, Seoul National University Hospital Healthcare System Gangnam Center and College of Medicine, Seoul 06236, Korea; ahreum.an@gmail.com (A.R.A.); belong@snu.ac.kr (B.C.); 3 Department of Surgery, Seoul National University Hospital, Seoul 03080, Korea; consultkang@hanmail.net; 4 Department of Breast and Endocrine Surgery, Hallym University Sacred Heart Hospital, Anyang 14068, Korea; nicizm@gmail.com; 5 Department of Surgery, Ewha Womans University College of Medicine, Seoul 07985, Korea; lovekkung@gmail.com; 6 Department of Surgery, Seoul National University Hospital and College of Medicine, Seoul 03080, Korea; kyueunlee@snu.ac.kr; 7 Department of Surgery, Seoul National University Boramae Medical Center, Seoul 07061, Korea; kevinjoon@naver.com

**Keywords:** quality of life, QoL, thyroidectomy, Short Form 12 questionnaire, SF-12

## Abstract

*Background and objectives:* The study assesses quality of life (QoL) in patients who underwent thyroidectomy compared to the general population. *Materials and Methods:* QoL data from post-thyroidectomy patients and individuals with no subjective health concerns, who had attended a routine health screening visit, were evaluated. QoL was assessed using the modified version of Korean Short Form 12 questionnaire (SF-12). Patients and controls were matched using the propensity score approach and a ratio of 1:4. *Results:* Data from a total of 105 patients and 420 controls were analyzed. For five SF-12 items, lower QoL was found in patients (*p* < 0.05). Multivariate analysis revealed that a follow-up duration of <1-year, female sex, and an age of >50 years were independent risk factors. No significant difference was found between controls and patients who were >1-year post-surgery. *Conclusions:* For specific SF-12 items, QoL was lower in post-thyroidectomy patients than in controls. No intergroup difference in QoL was found >1-year post-surgery.

## 1. Introduction

Quality of life (QoL) refers to perceived well-being, and encompasses the evaluation of physical health, and physical, social, and psychological functioning [1]. Over the past 30 years, research into the measurement and implications of QoL has shown a pronounced increase [2]. However, available QoL research has focused on a limited number of diseases, such as breast and colorectal cancer [3,4]. Factors with a reported potential impact on QoL mainly include surgical intervention.

Thyroidectomy is the treatment of choice in thyroid cancer patients, and the prevalence of thyroid cancer has shown a gradual increase over the past two decades [5,6,7]. Thyroid surgery is also indicated in certain forms of benign thyroid disorder, such as nontoxic goiter and Graves’ disease [8,9]. Although thyroidectomy is a universal operation, few studies to date have investigated the impact of thyroidectomy on QoL. Previous studies from the Canada and Singapore showed that the QoL scores of patients who had undergone thyroidectomy were significantly lower than those in individuals from the general population, as measured using the Short Form 36 questionnaire (SF-36) [10,11]. However, neither study used the propensity score approach to match patients and individuals from the general population, and the SF-36 data for the general population used in the Singapore analyses were obtained from another study [11]. By contrast, a Swedish study found no difference in QoL between patients who had undergone thyroidectomy with differentiated thyroid cancer and matched controls from the general population [12]. Post-thyroidectomy QoL has become an important issue in routine surgical practice. However, no consensus has been reached concerning the degree of QoL impairment experienced by patients following thyroidectomy, and varying causes for the reduction in QoL have been proposed. To date, QoL research in thyroidectomy patients has generated conflicting data concerning the influence of age and sex, as well as the time required to regain a QoL similar to that observed in the general population [13].

The aim of the present single-center, retrospective study was to compare QoL between patients who had undergone thyroidectomy and matched individuals from the general population with no subjective health concerns who had attended a routine health screening examination. QoL was evaluated using the modified version of the Korean Short Form 12 questionnaire (SF-12). In addition, analyses of individual SF-12 items were performed to identify factors associated with QoL in post-thyroidectomy patients.

## 2. Materials and Methods

### 2.1. Patient Group

Between September and December 2014, all patients attending the surgical outpatient clinic following thyroidectomy at the Seoul National University Hospital were assessed by a single trained interviewer. All patients were asked to complete the modified version of the Korean SF-12. In addition, data were collected on sociodemographic factors (i.e., age, sex, education status, monthly household income, and employment status) and health behavior (i.e., smoking and alcohol consumption). Inclusion criteria for the present analyses were post-thyroidectomy patients with complete SF-12. In the case of benign goiter, thyroid lobectomy was performed when the goiter was confined to one lobe, and total thyroidectomy was performed in case of both lobes. In thyroid cancer, intrathyroidal patients with no metastasis and confined to one side underwent thyroid lobectomy with prophylactic central lymph node (LN) dissection. In the case of bilateral lobe cancer, total thyroidectomy and prophylactic LN dissection were performed.

### 2.2. Control Group

Controls were drawn from individuals from the general population who had attended a routine health screening assessment at the Health Promotion Center of the Seoul National University Hospital between 2010 and 2011. All individuals were asked to complete the modified version of the Korean SF-12. In addition, data were collected on sociodemographic factors (i.e., age, sex, marital status, education status, monthly household income, and employment status), health behavior (i.e., smoking and alcohol consumption) and any history of cancer. Inclusion criteria for the present analyses were age ≥ 20 years, no history of cancer, completion of all items on the SF-12, and no subjective health concerns.

### 2.3. Propensity Score Matching

To reduce potential confounding effects, cases and controls were matched using the propensity score approach [14]. Propensity score matching was initially performed for age and sex only, using a patient to control ratio of 1:4. This revealed differences between patients and controls in terms of smoking, alcohol consumption, and monthly income. Propensity score matching was therefore repeated using a patient to control ratio of 1:4 and the factors age, sex, smoking, alcohol consumption, and monthly income.

### 2.4. Measurement of QoL

The SF-12 is derived from the SF-36, which is one of the most widely used generic instruments for the evaluation of QoL worldwide [15]. The SF-12 is a multipurpose, short form health survey that includes 12 items from the SF-36 [15]. Due to its brevity and the fact that it measures numerous aspects of health status, the SF-12 has become the instrument of choice in surveys of QoL in the general population. Table 1 shows the items and scoring system of the modified version of the Korean SF-12. Item SF-1 addresses general health (GH) and is scored using a 1 to 4 ordinal scale. High numbers indicate a positive status and lower numbers indicate a negative status (e.g., 1 = poor, 4 = excellent). Items SF-2 and SF-3 address physical functioning (PF) and are also scored using a 1 to 4 ordinal scale. Items SF-4 and SF-5 address role physical (RP) and are scored using a binary no (score = 1) or yes (score = 2) response. Items SF-6 and SF-7 address role emotional (RE) and are also scored using a binary no (score = 1) or yes (score = 2) response. For binary scales, a score of 1 indicates “limited” and a score of two indicates “not limited”. Item SF-8 addresses bodily pain (BP) and is scored using a 1 to 4 ordinal scale. Item SF-9 addresses mental health (MH) and is scored using a 1 to 6 ordinal scale. Item SF-10 addresses vitality (VT) and is scored using a 1 to 6 ordinal scale. Item SF-11 addresses MH and is scored using a 1 to 6 ordinal scale. Item SF-12 addresses social functioning (SF) and is scored using a 1 to 6 ordinal scale. In summary, the physical aspects of QoL are addressed using items SF-1, SF-2, SF-3, SF-4, SF-5, and SF-8, while the mental aspects of QoL are addressed using items SF-6, SF-7, SF-9, SF-10, SF-11, and SF-12. To determine the impact on QoL of follow-up duration, patients were divided into two subgroups on the basis of the length of outpatient surgical follow-up: (i) <1 year and (ii) ≥1 year. To ensure that the configuration of the two groups was similar, each subgroup was compared to the control group using the X2-test and the Student’s *t*-test.

### 2.5. Statistics

Categorical variables were assessed using the X2-test. Ordinal variables were treated as continuous variables and analyzed using the Student’s *t*-test. To estimate the influence of sociodemographic factors and clinical variables on overall QoL in patients, logistic regression analyses were performed. For the purposes of these analyses, the ordinal scores for items SF-11 and SF-12 (i.e., 1 to 6) were changed to binary format, i.e., scores 1 to 3 were changed to 0, and scores 4 to 6 were changed to 1. Significant variables were included in a univariate logistic regression model, and a subsequent multivariate analysis was performed. Significance was set at *p* < 0.1 for univariate analyses, and *p* < 0.05 for multivariate analyses. Age and sex were included in the multivariate analysis irrespective of significance level since these are important risk factors in thyroid cancer [16]. All analyses were performed using the SPSS statistical package for Windows (version 22.0).

### 2.6. Compliance with Ethical Standards

The study protocol was approved by the Institutional Review Board of the Seoul National University Hospital (H-1401-060-550). All study procedures were performed in accordance with the guidelines of the 2013 revision of the Declaration of Helsinki.

## 3. Results

### 3.1. Sociodemographic Characteristics of Patients and Controls

**Figure 1:**
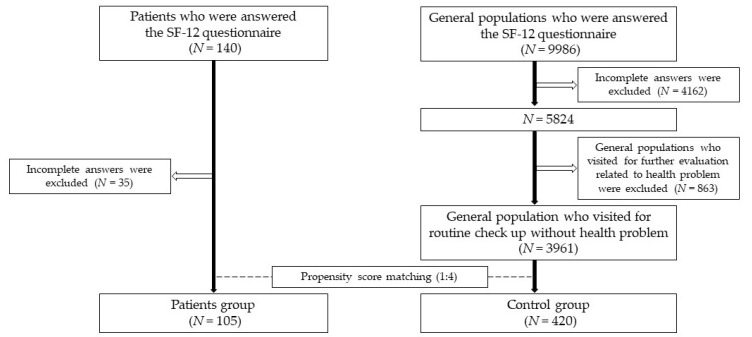
Study design with patients and controls. SF: Short Form; *N*: number.

The establishment of the study cohort is summarized in Figure 1. SF-12 data of patients were available for a total of 140 patients. Of these, 105 patients completed the SF-12 in full. The data of these 105 patients were included in the analyses. SF-12 data were available for a total of 9986 attendees of the health screening visit. For 4162 individuals, the SF-12 responses were incomplete and the data of these individuals were therefore excluded. Of the remaining 5824 individuals, 863 had attended the checkup appointment due to subjective health concerns, and their data were therefore excluded. The sociodemographic characteristics of the study cohort are shown in Table 2. No significant intergroup differences were observed. The mean age was 49.2 ± 12.6 years in the patient group, and 49.9 ± 13.1 years in controls. The proportion of females was 83.8% in the patient group and 85.0% in the control group. The percentage for smoking was 15.2% in patients and 12.4% in controls. The percentage for alcohol consumption was 27.6% in the patient group and 34.0% in the control group. A total of 77.7% of patients and 75.2% of controls were married. Education beyond high school was reported in 86.5% of patients and 86.2% of controls. A monthly household income of more than 4 million won was reported in 47.5% of patients and 53.0% of controls. A total of 53.3% of patients and 46.5% of controls were employed. The clinical characteristics of the patient group are shown in the lower part of Table 2. The mean follow-up period was 20.3 months (range, 0.3–205.0). A total of 83 patients (79.0%) underwent total thyroidectomy and 22 patients (21.0%) underwent lobectomy. At the time of surgery, 16 patients (17.6%) had presented with benign disease. The remaining 75 patients (82.4%) had presented with malignant thyroid disease: stage I or II, *n* = 42 (56.0%) and stage III and IV, *n* = 33 (44.0%).

### 3.2. Comparison of QoL between Patients and Controls

**Figure 2:**
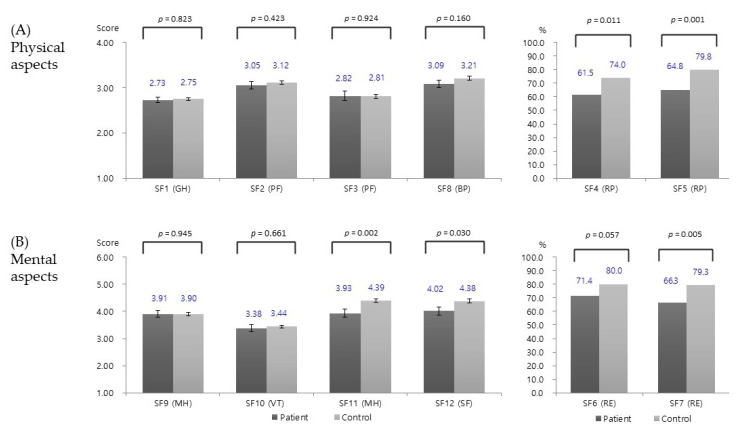
Physical and mental aspects of the modified Korean Short Form 12 questionnaire (**A**) Physical aspects (**B**) Mental aspects. GH: general health; PF: physical functioning; BP: bodily pain; RP: role physical; MH: mental health; VT: vitality; SF: social functioning; RE: role emotional.

Results for the physical aspects of QoL are shown in Figure 2A. A high score means good condition, and a low score means negative result. For item SF-4 (RP), there were significantly more good conditions in controls for reported physical difficulties compared to patients (mean: 61.5% in patients, 74.0% in controls; *p* = 0.011). For item SF-5 (RP), there were also significantly more good situations in controls for reported physical difficulties than in patients (mean: 64.8% in patients, 79.8% in controls; *p* < 0.001). No significant intergroup difference was found for SF-1 (GH), SF-2 (PF), SF-3 (PF), or SF-8 (BP).

Results for the mental aspects of QoL are shown in Figure 2B. For item SF-7 (RE), there were significantly more good conditions in controls than in patients (mean: 66.3% in patients, 79.3% in controls; *p* = 0.005). For items SF-11 (MH) and SF-12 (SF), there were significantly more good conditions in controls for mental difficulties than in patients (*p* = 0.002 and *p* = 0.03, respectively). No significant intergroup difference was found for item SF-9 (MH) or item SF-10 (VT).

### 3.3. Factors Associated with QoL Impairment in Patients

Logistic regression analysis was performed using those SF-12 items for which intergroup differences had been observed, i.e., SF-4, SF-5, SF-7, SF-11, and SF-12 (Table 3).

For the physical aspects of QoL, logistic regression analysis was performed using RP items SF-4 and SF-5. In the univariate analysis of SF-4 (RP), sex (*p* = 0.054) and alcohol consumption (*p* = 0.021) were significant. In the multivariate analysis of SF-4, no factor was significant. In the univariate analysis of SF-5 (RP), sex (*p* = 0.027), alcohol consumption (*p* = 0.054), employment status (*p* = 0.053), and follow-up duration (*p* = 0.001) were significant. In the multivariate analysis of SF-5, a follow-up duration of ≤1 year was the only significant factor (adjusted odds ratio (aOR) = 4.726 (95% CI), *p* = 0.001).

For the mental aspects of QoL, logistic regression analysis was performed using items SF-7 (RE), SF-11 (MH), and SF-12 (SF). In the multivariate analysis of SF-7 (RE), female sex was associated with a higher risk of mental difficulty than male sex (aOR = 0.122 (95% CI), *p* = 0.048). In the multivariate analysis, age was identified as a risk factor for SF-11 (MH). Here, mental difficulties were reported more frequently in patients aged ≥ 50 years compared to patients aged < 50 years (aOR = 0.283 (95% CI), *p* = 0.01). Multivariate analysis revealed no risk factors for SF-12 (SF).

### 3.4. Patients with Less Than 1 Year Follow-Up vs. More Than 1 Year Follow-Up

Table 4 shows the analysis of the impact on individual SF-12 items of follow-up duration; for patients with less than 1 year follow-up and more than 1 year follow-up values of 48 and 56 were obtained, respectively. To confirm that each subgroup had a similar configuration, both were compared to the control group. No significant difference in sociodemographic characteristics was found between the two follow-up duration subgroups and the control group. For patients with <1 year of follow-up, a significant difference to controls was observed for seven SF-12 items: SF-4 (*p* = 0.009), SF-5 (*p* < 0.001), SF-6 (*p* = 0.033), SF-7 (*p* = 0.008), SF-8 (*p* = 0.007), SF-11 (*p* = 0.001), and SF-12 (*p* = 0.004). No statistical difference to controls was found for patients with ≥1 year of follow-up.

## 4. Discussion

In recent years, QoL in thyroid patients has become an important subject since the incidence of thyroid disease is increasing and the post-surgical maintenance of QoL is an important consideration for both patients and surgeons. Limitations of previous studies included a lack of: (i) adequate case–control matching for sociodemographic factors and (ii) screening for health status in controls. The present study addressed both of these limitations of previous studies. The analysis of health–behavior characteristics is also a strength of the present study. The aim of the present study was to compare QoL between patients who had undergone thyroidectomy and individuals from the general population with no subjective health problems. Patients and controls from a single center were matched for sociodemographic factors and health–behavior characteristics, using propensity score matching.

In the comparison of patients and controls, significant differences were observed for five SF-12 items: (i) physical aspect items SF-4 and SF-5 (*p* = 0.011 and *p* = 0.001, respectively) and (ii) mental aspect items SF-7, SF-11, and SF-12 (*p* = 0.005, *p* = 0.002, and *p* = 0.03, respectively). Thus, for seven SF-12 items, no intergroup differences were observed. The decrease in QoL observed in the present analyses is consistent with previous reports [10,11]. To determine risk factors for the observed decrease in QoL among patients, logistic regression analyses were performed. These revealed that a follow-up period of <1 year, age >50 years, and female sex were risk factors for reduced QoL following thyroidectomy in selective items. First, a follow-up duration of <1 year was associated with a lower QoL than a follow-up duration of ≥1 year for specific SF-12 items. In contrast, a previous study from Sweden found no association between post-thyroidectomy QoL and follow-up duration [12]. The result in the Swedish study may have been attributable to the fact that the mean period of follow-up was 10 years. On the other hand, the mean follow-up period of the present cohort was relatively short (around 20 months). Second, the present study also found an association between age and a reported decline in mental health, particularly among patients aged > 50 years. In the 12 month post-operative period, screening for psychiatric symptoms may thus be helpful in patients aged 50 years and above. Finally, female sex was identified as a risk factor for reduced QoL post-thyroidectomy in a specific item. To our knowledge, the present study is the first to suggest this association.

Additional analyses were performed to determine the effect of follow-up duration on QoL. These showed that impairments in QoL were more frequent in patients with a follow-up duration of <1 year compared to patients with a follow-up period of >1 year. In a proportion of patients, this may have been attributable to post-operative complications such as hypocalcemia, vocal cord paralysis, or hematoma during the 1 year post-operative period. In thyroid cancer patients, post-operative radioactive iodine treatment may be implicated in the observed decrease in QoL [17]. To our knowledge, the present study is the first investigation of the post-operative follow-up period in thyroidectomy patients to demonstrate a recovery of QoL after 1 year. This finding suggests that during the initial 1 year follow-up period, psychiatric consultation or supportive care may be helpful for patients with low physical or mental aspect scores. Further studies are warranted to identify the specific risk factors for reduced QoL within 1 year of thyroidectomy.

A post-operative decrease in QoL with subsequent recovery has also been reported following breast and colorectal surgery. For example, a case–control study involving 542 patients with breast cancer demonstrated a minimum QoL score at 6 months post-surgery with recovery after 1 year [18]. The authors proposed that the observed reduction in QoL at 6 months may have been attributable to endocrine therapy and chemotherapy [18]. In a study of 100 colorectal cancer patients, overall QoL was comparable to that of subjects from the general population 1 year post-surgery [19]. A plausible hypothesis is that QoL decreases in the early post-operative period as a result of acute complications, pain, or the impact of additional surgery and/or therapy [20].

The present study had three main limitations. First, a modified version of the validated Korean SF-12 was used. In general, the physical and the mental component scores of the SF-12 were analyzed. However, for the purposes of the present study, each of the 12 items were analyzed individually. Second, no data were available from patients concerning past medical history, radioactive iodine therapy, or post-surgical complications such as delayed wound healing, hypocalcemia, or vocal cord paralysis. Further investigations are warranted to determine whether these factors lead to an impairment in QoL post-thyroidectomy, as this would facilitate clinical management. Third, detailed studies according to the classification of surgical methods such as total thyroidectomy and thyroid lobectomy are insufficient. This content requires further research.

In conclusion, a follow-up duration shorter than 1-year, female sex, and age over 50 years is independent risk factors among patients with thyroidectomy. Furthermore, the present study demonstrates that in a proportion of patients, QoL was reduced following thyroidectomy. However, QoL returned to the level reported in matched controls from the general population 1 year after surgery.

## Figures and Tables

**Table 1:** Modified Korean version of the Short Form 12 questionnaire (SF-12).

SF	Items (Abbreviation)	Aspect	Types of Variables	Answers	Question
SF-1	General Health (GH)	Physical	Ordinal	1 (Poor) ⇔ 4 (Excellent)	In general, would you say your health is
SF-2	Physical Functioning (PF)	Physical	Ordinal	1 (Limited a lot) ⇔ 4 (Not limited at all)	Limitation in moderate activities
SF-3	Physical Functioning (PF)	Physical	Ordinal	1 (Limited a lot) ⇔ 4 (Not limited at all)	Limitation in climbing several flights of stairs
SF-4	Role Physical (RP)	Physical	Binary	1 (Limited), 2 (Not limited)	Accomplished less than you would like due to physical problem (past 4 weeks)
SF-5	Role Physical (RP)	Physical	Binary	1 (Limited), 2 (Not limited)	Were limited in the kind of work or other activities due to physical problem (past 4 weeks)
SF-6	Role Emotional (RE)	Mental	Binary	1 (Depressed), 2 (Not depressed)	Accomplished less than you would like due to emotional problems (past 4 weeks)
SF-7	Role Emotional (RE)	Mental	Binary	1 (Depressed), 2 (Not depressed)	Did not do work or other activities as carefully as usual due to emotional problems (past 4 weeks)
SF-8	Bodily Pain (BP)	Physical	Ordinal	1 (Extreme pain) ⇔ 4 (Not at all)	How much did pain interfere with your normal work? (past 4 weeks)
SF-9	Mental Health (MH)	Mental	Ordinal	1 (Depressed) ⇔ 6 (Not depressed)	Have you felt calm and peaceful? (past 4 weeks)
SF-10	Vitality (VT)	Mental	Ordinal	1 (Depressed) ⇔ 6 (Not depressed)	Did you have a lot of energy? (past 4 weeks)
SF-11	Mental Health (MH)	Mental	Ordinal	1 (Depressed) ⇔ 6 (Not depressed)	Have you felt downhearted and blue? (past 4 weeks)
SF-12	Social Functioning (SF)	Mental	Ordinal	1 (Depressed) ⇔ 6 (Not depressed)	How much of the time has your physical health or emotional problems interfered with your social activities?

SF: Short Form.

**Table 2:** Demographic and clinical characteristics of patients and controls. Cases and controls were matched for age, sex, smoking, alcohol consumption, and monthly income using the propensity score approach.

		Patient Group (*n* = 105)		Control Group (*n* = 420)		
		*n*	%	*n*	%	*p* Value
Age	Mean ± SD	49.2 ± 12.6		49.9 ± 13.1		0.609
Sex	Male	17	16.2	63	15.0	0.761
	Female	88	83.8	357	85.0	
Smoking	No	89	84.8	368	87.6	0.435
	Yes	16	15.2	52	12.4	
Alcohol consumption	No	76	72.4	277	72.4	0.209
	Yes	29	27.6	143	34.0	
Spouse	Yes	80	77.7	306	75.2	0.599
	No	23	22.3	101	24.8	
Education	≥College above	50	48.1	244	58.1	0.065
	≤High School	54	51.3	176	41.9	
Monthly household income (KRW)	≥4,000,000	47	47.5	215	53.0	0.328
	<4,000,000	52	52.5	191	47.0	
Employed	Yes	56	53.3	191	46.5	0.209
	No	49	46.7	220	53.5	
F/U duration	Mean ± SD (min–max) (mo)	20.3 ± 29.5 (0.3–205.0)				
Op type	Total thyroidectomy	83	79.0			
	lobectomy	22	21.0			
Benign vs. Malignant	Malignancy (I and II/III and IV)	75 (42/33)	82.4 (56/44)			
	Benign	16	17.6			

SD: standard deviation; KRW: South Korea won rate; F/U: follow-up; mo: month; I: stage I; II: stage II; III: stage III; IV: stage IV.

**Table 3:** Multivariate analysis to determine risk factors for decreased QoL post-thyroidectomy.

		SF-4 (aOR(95% CI))	SF-5 (aOR(95% CI))	SF-7 (aOR(95% CI))	SF-11 (aOR(95% CI))	SF-12 (aOR(95% CI))
Age	<50 years (ref) vs. ≥50 years	0.485 (0.210–1.122)	0.957 (0.383–2.389)	1.399 (0.592–3.305)	0.283 (0.109–0.737)	/
Sex	Male (ref.) vs. Female	0.332 (0.084–1.314)	0.284 (0.052–1.545)	0.122 (0.015–0.983)	0.990 (0.283–3.467)	/
Alcohol	Yes (ref) vs. No and Past	2.723 (0.959–7.734)	1.731 (0.569–5.265)	2.382 (0.781–7.262)		/
Employed	Yes (ref) vs. No	/	0.441 (0.170–1.145)	/	2.531 (0.962–6.657)	/
F/U duration	<1 year (ref) vs. ≥1 year	/	4.726 (1.862–11.999)	/	2.2 (0.902–5.369)	/
Op type	Lobectomy (ref) vs. Total thyroidectomy	/	/	/	2.784 (0.954–8.124)	/

QoL: quality of life; aOR: adjusted odds ratio; CI: confidence interval; ref: reference.

**Table 4:** Analysis of changes on individual SF-12 items 1 year before and after follow-up duration.

	Patients <1 Year (*n* = 48) vs. Controls (*n* = 420)		Patients ≥1 Year (*n* = 56) vs. Controls (*n* = 420)	
		*p* Value		*p* Value
SF-1 (GH)	2.77 ± 0.074 vs. 2.75 ± 0.028	0.770	2.70 ± 0.095 vs. 2.75 ± 0.028	0.566
SF-2 (PF)	2.94 ± 0.109 vs. 3.12 ± 0.038	0.129	3.14 ± 0.121 vs. 3.12 ± 0.038	0.832
SF-3 (PF)	2.79 ± 0.143 vs. 2.81 ± 0.047	0.916	2.84 ± 0.146 vs. 2.81 ± 0.047	0.817
SF-4 (RP)	56.3% vs. 74.0%	0.009	66.1% vs. 74.0%	0.206
SF-5 (RP)	47.9% vs. 79.8%	<0.001	78.9% vs. 79.8%	0.886
SF-6 (RE)	66.7% vs. 80.0%	0.033	75.4% vs. 80.0%	0.424
SF-7 (RE)	62.5% vs. 79.3%	0.008	69.6% vs. 79.3%	0.101
SF-8 (BP)	2.88 ± 0.132 vs. 3.21 ± 0.039	0.007	3.26 ± 0.102 vs. 3.21 ± 0.039	0.631
SF-9 (MH)	3.83 ± 0.200 vs. 3.90 ± 0.061	0.712	3.98 ± 0.171 vs. 3.90 ± 0.061	0.663
SF-10 (VT)	3.40 ± 0.175 vs. 3.44 ± 0.057	0.813	3.37 ± 0.185 vs. 3.44 ± 0.057	0.679
SF-11 (MH)	3.71 ± 0.223 vs. 4.39 ± 0.065	0.001	4.12 ± 0.191 vs. 4.39 ± 0.065	0.157
SF-12 (SF)	3.91 ± 0.213 vs. 4.38 ± 0.073	0.004	4.11 ± 0.218 vs. 4.38 ± 0.073	0.202

GH: general health; PF: physical functioning; BP: bodily pain; RE: role emotional; RP: role physical; MH: mental health; VT: vitality; SF: social functioning.
